# Attitudes towards opportunistic salpingectomy among patients planned to undergo non-gynecologic intra-abdominal surgery

**DOI:** 10.1016/j.gore.2025.102000

**Published:** 2025-12-02

**Authors:** Monali S. Ardeshna, Lauren Dori, Benjamin Margolis

**Affiliations:** a Albany Medical College, 47 New Scotland Avenue, Albany NY 12208, United States; b Albany Medical Center, Department of Obstetrics and Gynecology. 43 New Scotland Avenue, Albany NY 12208, United States

**Keywords:** Opportunistic salpingectomy, Ovarian cancer, Cancer prevention, Women’s health

## Abstract

**Objective:**

To evaluate the knowledge of and interest in opportunistic salpingectomy (OS) in patients undergoing non-gynecologic abdominal surgery.

**Methods:**

English-speaking patients aged 18 or older with fallopian tubes who were scheduled for upcoming abdominal general, colorectal, or bariatric surgery were approached. A phone-administered questionnaire was used to collect demographics, clinical history, knowledge of OS, and likelihood to undergo OS if offered. Chi-square tests, Fisher’s exact tests, and odds ratios were calculated in R.

**Results:**

153 patients were contacted, of which 68 were eligible and agreed to participate (44.4 % response rate). Of the 68 participants, 60 (88.2 %) were considered OS candidates (not interested in future fertility). The average age was 58, and 97 % of respondents self-identified as White. Forty-five respondents (75.0 %) were post-menopausal, 11 (18.3 %) had undergone a hysterectomy, and 16 (26.7 %) had undergone permanent contraception. Only 12 (20.0 %) knew OS reduces ovarian cancer risk. Among OS candidates, 15 (25.0 %) were likely to undergo OS during their upcoming surgery if offered. Predictors of likelihood to undergo OS during surgery include awareness of OS (50.0 % vs 19.0 %, OR 4.33, 95 % CI: 1.13–16.61, p = 0.025), family history of ovarian cancer (66.7 % vs 20.4 %, OR 7.82, 95 % CI: 1.26–48.35, p = 0.030), and interest in permanent contraception (100 % vs 25 %, p = 0.044).

**Conclusion:**

Among patients with fallopian tubes undergoing non-gynecologic abdominal surgery, most were appropriate candidates for OS. Prior awareness of OS, family history of ovarian cancer, and interest in permanent contraception were associated with a greater interest in OS.

## Highlights

• Only 20% of patients were aware that OS lowers ovarian cancer risk.
• One in four patients scheduled for non-gynecologic surgery would accept OS at the time of their upcoming procedure.
• Higher OS interest was found in pre-menopausal respondents with interest in permanent contraception.
• Respondents with prior awareness of OS and a family history of ovarian cancer had higher odds of OS interest.
• 12.1% of those who denied OS interest stated they needed more information on OS, suggesting a need for patient education.

## 1 Introduction

The most widely accepted theory of ovarian cancer pathogenesis supports the fallopian tube epithelium as the site of origin of most if not all epithelial serous ovarian cancers (Kurman and IeM, 2010, Kurman and IeM, 2016, Erickson et al., 2013, Crum, 2009, Kindelberger et al., 2007). Serous tubal intraepithelial carcinomas have been identified as a precursor lesion to serous carcinoma and follow a predictable oncogenesis that stems from a near-universal driver TP53 mutation that is seen in 95 % of serous cancers (Kindelberger et al., 2007, Kuhn et al., 2012, Hanley et al., 2015). Opportunistic salpingectomy (OS) refers to the removal of the entire fallopian tube to reduce the risk of ovarian cancer during gynecologic surgery (Opportunistic salpingectomy as a strategy for epithelial ovarian cancer prevention. ACOG. (n.d.). Retrieved May 1, 2023). Retrospective data strongly support that OS is associated with reduced incidence of ovarian cancer (Hanley et al., 2022, Falconer et al., 2015). This has led to the widespread adoption of OS during gynecologic surgery. As the benefits of OS would also apply to patients undergoing non-gynecologic surgery, there has been interest in adopting OS in this population. OS has been predicted to reduce ovarian cancer deaths by nearly 7 % if performed during elective non-gynecologic surgeries (Hughes et al., 2022). Cost effectiveness has also been suggested for OS during cholecystectomy and appendectomy (Matsuo et al., 2023 May 1, Hughes et al., 2019). Few studies have examined feasibility and implementation of OS for non-gynecologic surgery.

An Austrian group first surveyed women scheduled to undergo cholecystectomy and found that 19 of 20 surveyed women would accept or likely accept salpingectomy with their planned cholecystectomy (Tomasch et al., 2018 Nov). The same group later demonstrated feasibility of OS at the time of cholecystectomy with a 60 % acceptance rate and a 93 % success rate among planned procedures (Tomasch et al., 2020). Given the relative paucity of data on acceptance of OS by patients, and no data to our knowledge on attitudes in patients planned to undergo abdominal procedures other than cholecystectomy, the primary objective of this study was to gauge interest, knowledge, and acceptance of OS among patients planned for non-gynecologic laparoscopic abdominal procedures.

## 2 Methods

English-speaking participants who were ≥ 18 years old and were scheduled for abdominal general, colorectal or bariatric surgery from September 2023 to September 2024 were invited to participate in this study. Eligible patients were identified from the institution’s surgical schedule and called prior to their procedure to evaluate interest in participation. Prospective participants were given oral information regarding the study’s purpose and underwent informed consent at the beginning of the interview. Patients were first questioned about prior salpingectomy and were excluded for participation in the study if they self-reported previous salpingectomy. The study protocol was approved by the local institutional review board.

A mixed methods research approach was used. Standardized scripted phone interviews were conducted by two interviewers to assess knowledge of and interest in OS in patients undergoing non-gynecologic abdominal surgery (Supplemental Material S1). The interviews took approximately 5 min each. Quantitative survey data were collected orally and were recorded by the interviewers, as were qualitative answers to open-ended questions. Demographic information collected included age, race, ethnicity, religion, and highest level of education. Race and ethnicity were considered relevant data points given the country’s history of unethical practices surrounding forced permanent contraception, which could lead to differences in acceptance of OS based on racial demographics. Religion was obtained given there are influences between some religious practices and contraception that could impact acceptance of OS. Information about the scheduled procedure was also collected from chart review. Obstetric and gynecologic (OBGYN) history included parity, menopausal status, history of hysterectomy, history of permanent contraception, interest in future fertility, and family history of ovarian cancer. To evaluate the knowledge of and interest in OS, patients were asked about awareness of OS, likelihood to choose OS at the time of their upcoming surgery, and the chances of regretting OS if completed. If a participant indicated they were not likely to elect for OS at the time of their upcoming surgery, they were asked to elaborate their reasoning. The open-ended responses were then coded and grouped into subcategories based on thematic alignment. Participants’ likelihood to elect for OS was collected on a 5-point Likert scale (extremely unlikely to extremely likely).

Data were analyzed using R with descriptive statistics and chi-square tests. Fisher's exact tests were instead used for comparisons involving expected counts less than five. Odds ratios and confidence intervals were calculated from contingency tables. Only candidates not interested in future fertility were included in the analysis since they would be appropriate OS candidates. P < 0.05 was considered significant. Race and ethnicity were not analyzed as potential modifiers of likelihood to choose OS and awareness of OS lowering the risk of ovarian cancer since not enough racial/ethnic variation was included in this study sample.

## 3 Results

**Fig. 1:**
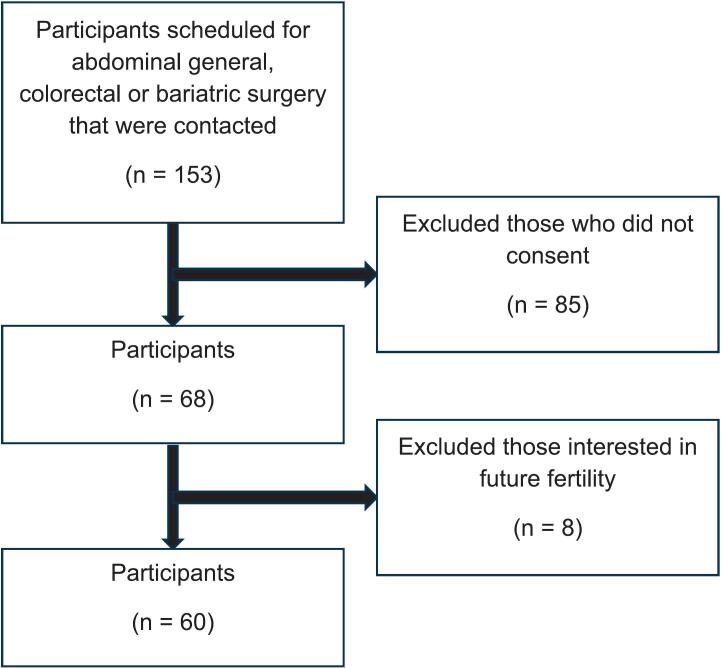
Patient inclusion flowchart.

153 patients were contacted, of which 68 initially agreed to participate (44.4 % response rate). Of the 68 participants, 60 (88.2 %) were not interested in future fertility and were considered OS candidates (Fig. 1).

Among 60 OS candidates, the average age was 58. Fifty-eight (96.7 %) self-identified as White and 2 (3.3 %) identified as Black. Participants were scheduled for the following procedures: 12 (20.0 %) were scheduled for hernia repair, 30 (50.0 %) for colorectal surgery, 7 (11.7 %) for cholecystectomy, 5 (8.3 %) for bariatric surgery, and 6 (10.0 %) for other abdominal surgery. Forty-five respondents (75.0 %) were post-menopausal, 11 (18.3 %) had undergone a hysterectomy, and 16 (26.7 %) had undergone permanent contraception. Among pre-menopausal patients, 3 (20.0 %) were interested in permanent contraception. Six respondents (10.0 %) had a family history of ovarian cancer. Patient characteristics are summarized in Table 1.

**Table 1:** Participant DEMOGRAPHICS and OBGYN history.

	*n*	%
**Age**		
18–49	14	23.3
50–64	27	45.0
65 and above	19	31.7
**Race**		
White	58	96.7
Black	2	3.3
**Ethnicity**		
Non-Hispanic	60	100.0
Hispanic	0	0.0
**Highest education level**		
High school or less	17	28.3
Some college	12	20.0
College graduate	19	31.7
Graduate school	12	20.0
**Religion**		
Religious	39	65.0
Not religious	21	35.0
**Type of abdominal surgery**		
Hernia repair	12	20.0
Colorectal surgery (colon, resection, ostomy)	30	50.0
Cholecystectomy	7	11.7
Bariatric surgery	5	8.3
Other	6	10.0
**Menopause**		
Pre-menopause	15	25.0
Post-menopause	45	75.0
**Interest in permanent contraception (of pre-menopausal patients)**		
Yes	3	20.0
No	12	80.0
**History of permanent contraception**		
Yes	16	26.7
No	44	73.3
**History of hysterectomy**		
Yes	11	18.3
No	49	81.7
**Family history of ovarian cancer**		
Yes	6	10.0
No	54	90.0
**Aware OS lowers risk of ovarian cancer**		
Yes	12	20.0
No	48	80.0
**Likely to undergo OS at time of non-gynecologic surgery**		
Yes	15	25.0
No	45	75.0

OBGYN, obstetric and gynecologic; OS, opportunistic salpingectomy.

**Fig. 2:**
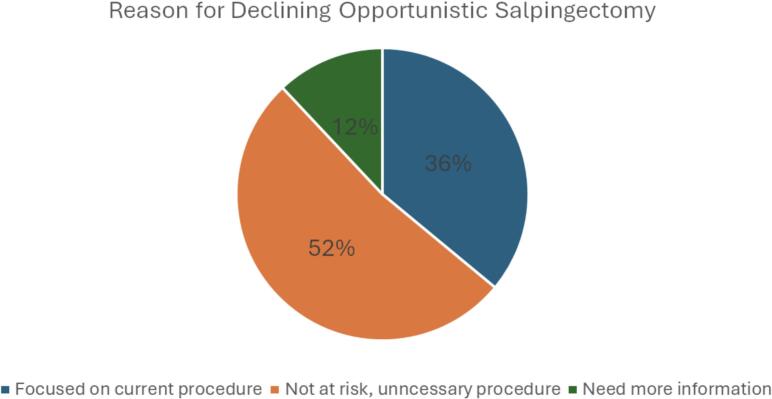
Reasons for Declining OS at time of surgery.

Among OS candidates, only 12 (20.0 %) were aware that OS reduces ovarian cancer risk. 15 (25.0 %) candidates were likely to undergo OS at the time of their upcoming surgery if offered (Table 1). Of the patients who indicated that they would be likely to undergo OS at the time of their upcoming surgery, all 15 (100.0 %) said they did not think they would experience regret. Of those who indicated they would not be interested in OS and were willing to elaborate why not, 12 (36.4 %) were stressed or focused on their current indicated procedure, 17 (51.5 %) did not think they were at risk or there was need for OS, and 4 (12.1 %) indicated they needed more information about OS before deciding (Fig. 2).

Age, race, education level, religiosity, and type of surgery were not associated with being likely to choose OS at the time of surgery or having prior awareness of OS (Table 2). Being likely to undergo OS at the time of surgery was more frequently reported among those who were aware of OS reducing ovarian cancer risk prior to the interview, those with a family history of ovarian cancer, and pre-menopausal respondents interested in permanent contraception. Specifically, 50 % of those aware of OS reducing ovarian cancer risk reported being likely to undergo the procedure compared to 19.0 % of those who were unaware (p = 0.025). 66.7 % of respondents with a family history of ovarian cancer reported being likely to undergo OS compared to 20.4 % of those without a family history (p = 0.030). Among pre-menopausal respondents interested in permanent contraception, 100 % reported being likely to undergo OS compared to 25 % of those not interested (p = 0.044). Corresponding odds ratios further quantified these associations. Respondents that were aware of OS reducing ovarian cancer risk had 4.33 times higher odds of being likely to undergo OS (95 % CI: 1.13–16.61). Respondents with a family history of ovarian cancer had 7.82 times higher odds of being likely to undergo OS (95 % CI: 1.26–43.35). An odds ratio could not be determined for pre-menopausal respondents interested in permanent contraception due to quasi-complete separation.

**Table 2:** Associations with Electing for OS at the Time of Surgery.

	Likely	Not likely	Total	*p*-value
**Age**^**†**^				
18–49	6 (42.9 %)	8 (57.1 %)	14	0.235
50–64	6 (22.2 %)	21 (77.8 %)	27	
65 and above	3 (15.8 %)	16 (84.2 %)	19	
Total	15	45	60	
**Education level**				
Less than college	7 (21.2 %)	26 (78.8 %)	33	0.870
College or more	8 (22.9 %)	27 (77.1 %)	35	
Total	15	53	68	
**Religion**				
Religious	7 (17.9 %)	32 (82.1 %)	39	0.086
Not religious	8 (38.1 %)	13 (61.9 %)	21	
Total	16	45	60	
**Type of abdominal surgery**^**†**^				
Hernia repair	4 (33.3 %)	8 (66.7 %)	12	0.219
Colorectal surgery	4 (13.3 %)	26 (86.7 %)	30	
Cholecystectomy	3 (42.9 %)	4 (57.1 %)	7	
Bariatric surgery	2 (40.0 %)	3 (60.0 %)	5	
Other	2 (33.3 %)	4 (66.7 %)	6	
Total	15	45	60	
**Menopause**^**†**^				
Pre-menopause	6 (40.0 %)	9 (60.0 %)	15	0.121
Post-menopause	9 (20.0 %)	36 (80.0 %)	45	
Total	15	45	60	
**Interest in permanent contraception (pre-menopausal patients)**^**†**^				
Yes	3 (100.0 %)	0 (0.0 %)	3	0.044*
No	3 (25.0 %)	9 (75.0 %)	12	
Total	6	9	15	
**History of permanent contraception**^**†**^				
Yes	3 (18.8 %)	13 (81.3 %)	16	0.738
No	12 (27.3 %)	32 (72.7 %)	44	
Total	15	45	60	
**History of hysterectomy**^**†**^				
Yes	4 (36.3 %)	7 (63.6 %)	11	0.442
No	11 (22.4 %)	38 (77.6 %)	49	
Total	15	45	60	
**Family history of ovarian cancer**^**†**^				
Yes	4 (66.7 %)	2 (33.3 %)	6	0.030*
No	11 (20.4 %)	43 (79.6 %)	54	
Total	15	45	60	
**Aware OS lowers risk of ovarian cancer**^**†**^				
Yes	6 (50.0 %)	6 (50.0 %)	12	0.025*
No	9 (18.8 %)	39 (81.3 %)	48	
Total	15	45	60	

*p < 0.05; † denotes the use of Fisher’s exact test instead of chi square; OS, opportunistic salpingectomy.

## 4 Discussion

OS at the time of gynecologic surgery has become widely accepted and routine in many countries (Ntoumanoglou-Schuiki et al., 2018, McAlpine et al., 2014). There is limited data regarding OS at the time of non-gynecologic surgery to our knowledge. Two studies by Tomasch et al have established the acceptability and feasibility of OS in patients scheduled for laparoscopic cholecystectomy (Tomasch et al., 2018 Nov, Tomasch et al., 2020). In their first study assessing patient interest, 60 % of women 45 years or older would accept concurrent salpingectomy at the time of their laparoscopic cholecystectomy. In their subsequent feasibility study, OS was successfully performed in 90 percent of laparoscopic cholecystectomy patients with limited additional operating time and complications. We add to the existing literature by demonstrating a 25 % acceptance of OS at the time of a wider array of abdominal non-gynecologic surgeries. Our results provide valuable information on factors that influence the decision to undergo OS, such as future fertility, prior knowledge of OS, and family history of ovarian cancer.

Our participants were less likely to accept OS than the 60 % acceptance rate by Tomasch et al. The inclusion of a larger variety of procedures likely introduced a more heterogeneous patient population, some of whom were planned for oncologic procedures and higher risk bariatric procedures with accompanying major lifestyle changes. The pre-occupation with a serious health condition may serve as a deterring factor to accept OS, as 36 percent of those who declined OS cited stress about their upcoming procedure as the reason for declining. Despite not reaching statistical significance, participants scheduled for cholecystectomy did have the highest rate of OS acceptance at 43 %, and those undergoing the likely highest risk procedures (colorectal surgery) had the lowest percentage of acceptance at 13 %. Another factor that likely influenced our lower acceptance rate was our recruitment method of calling patients who were already scheduled for surgery. Introduction of the concept of OS by the patient’s surgeon at the time of booking would likely improve trust in and acceptance of the procedure.

Identifying factors that influence acceptance of OS can inform future efforts to bring OS to patients undergoing non-gynecologic abdominal surgery. Acceptance of OS at the time of surgery was more frequently reported among those who were already aware that OS reduces ovarian cancer risk. With only 20 % of OS candidates reporting awareness of OS, there is an opportunity for patient education which would likely impact OS uptake if offered. In a study assessing patient and surgeon attitudes towards OS in non-gynecologic surgery, patients viewed the procedure favorably but emphasized the importance of raising OS awareness (Tymm et al., 2024). Most general surgeons surveyed in this study were not aware themselves of the serous tubal intraepithelial carcinoma hypothesis or OS offering cancer risk‐reduction. After education, most participants were interested in incorporating OS into their practice. These data suggest a lack of both patient and provider education about the practice of OS.

Unsurprisingly, those with a family history of ovarian cancer and those interested in permanent contraception were especially receptive of OS. As we think about incorporating OS into the clinical encounter of patients in a non-gynecologic surgery clinic, screening for patients interested in permanent contraception or with a family history of ovarian cancer could help offices reach those most likely to undergo the procedure.

OS during non-gynecologic surgery is currently an underutilized strategy for cancer prevention (Cathcart et al., 2023 Aug 11). Out of all hospital admissions involving bilateral salpingectomy from 2016 to 2020 in the US, only 0.05 percent were performed during non-gynecologic surgery. OS during non-gynecologic surgery would be estimated to prevent between 3600 and 5800 deaths from ovarian cancer per year nationally if attempted in 60 % of the non-gynecologic surgeries performed, with an estimated success rate of 90 %, assuming a reduction in lifetime ovarian cancer risk between 40–65 % (Falconer et al., 2015, Tomasch et al., 2020, Cathcart et al., 2023 Aug 11). Despite our lower rate of acceptance of OS for patients undergoing a wide array of non-gynecologic abdominal procedures, there remains significant interest in the procedure even when introduced by a cold call shortly before a scheduled procedure.

Limitations of this study include a lack of racial and ethnic diversity. Nearly all participants (97 %) identified as White, which restricts the generalizability of the findings. Patient perspectives may differ across more diverse populations. Undoubtedly, introduction of OS to more diverse populations could be met with hesitation given our country’s history of forced sterilization of minority populations (Reilly, 2015). Increasing diversity of the sample would be important in future studies. Additionally, English proficiency was an inclusion criterion for our sample group. This further narrows the applicability of the results, as it excludes individuals with limited English proficiency who may face unique structural barriers or cultural considerations that could shape their opinion of OS. Due to logistical constraints, we were not able to introduce the idea of OS by the patients’ trusted surgeons at the time of a clinical encounter, which would be the ideal way to educate patients about OS. As with any survey, there can be participation bias resulting in more willingness to engage with the topic of OS among those willing to participate in the survey. Recall bias and accuracy of patients’ knowledge of prior salpingectomy could also lead to inaccurate patient reporting, however the expressed interest in removing tubes that are thought to be present is still valuable information even if the patient incorrectly reported no prior salpingectomy but in fact did have one. Strengths include the prospective nature of the data collection, richness of historical data to evaluate predictors of acceptance of OS, and inclusion of patients undergoing multiple types of intra-abdominal procedures. Although we did collect some qualitative data on reasons for declining OS, more in-depth qualitative interview data would be useful to further characterize barriers and evaluate the most effective ways to introduce and explain the topic of OS to potential candidates.

There are many logistical considerations that make widespread adoption of OS at the time of non-gynecologic surgery a significant hurdle and are outside the scope of this study. These include development of protocols, availability of gynecologic surgeons to perform the procedure, training of non-gynecologic surgeons to perform the procedure, surgeon credentialing, insurance reimbursement, pathology reimbursement and triage of abnormal pathology results to name a few. Despite these hurdles, given the fact that there are no effective screening strategies for ovarian cancer and most patients present with advanced stage disease, focusing on prevention is key. OS at the time of non-gynecologic surgery is also predicted to be cost effective, with OS during appendectomies being the most cost-effective (Dilley et al., 2017). We show that even with late introduction of OS into surgical care, up to 25 % would consider the procedure in addition to their primary surgery. This percentage may improve with earlier introduction of the topic and increased patient education.

## CRediT authorship contribution statement

**Monali S. Ardeshna:** Writing – review & editing, Writing – original draft, Visualization, Methodology, Investigation, Formal analysis, Data curation. **Lauren Dori:** Writing – review & editing, Writing – original draft, Visualization, Methodology, Investigation, Formal analysis, Data curation, Conceptualization. **Benjamin Margolis:** Writing – review & editing, Writing – original draft, Supervision, Methodology, Investigation, Conceptualization.

## Declaration of competing interest

The authors declare that they have no known competing financial interests or personal relationships that could have appeared to influence the work reported in this paper.
