# Supplementary material for: Attitudes towards opportunistic salpingectomy among patients planned to undergo non-gynecologic intra-abdominal surgery
Source: Gynecol Oncol Rep. 2025 Dec 2;62:102000. doi: 10.1016/j.gore.2025.102000 (PMC12720371; doi:10.1016/j.gore.2025.102000)
Supplement: Supplementary Data 1 [file mmc1.docx]

S1. Survey Instrument

“Hello (participant) . . . My name is (name) and I’m calling from Albany Medical Center. I am part of a research team in the OBGYN department and we are doing a research project about the removal of the fallopian tubes at the time of non-gynecologic surgery. We were told you are getting scheduled for a surgery involving the intestines. Are you open to answering a few questions either now or at a later date about the idea of fallopian tube removal? There are 12 questions and this is expected to take 5-10 minutes.”

*(If participant wishes, set up an alternative time to discuss project)*

*(If participant states fallopian tubes are already removed, note this and state that this is all the information that would be needed, conclude the call)*

*(If participant is open, then provide brief consent)*

This project is a de-identified survey which means none of your personal identifying information will be recorded or used. After this phone interview your participation in the study will be over and we will not contact you again. Are you okay if we proceed?

*(If okay, then continue)*

I’m going to start by reading a short statement to describe why we are asking these questions:

“Removal of the fallopian tubes at the time of gynecologic surgery has been shown to reduce the chance of developing ovarian cancer in the future. This is recommended when undergoing procedures on other gynecologic organs, and we are interested in extending this benefit to people who are undergoing different types of surgery. The following questions are intended to hear your thoughts and opinions on the topic, and do NOT mean that any additional surgical procedures will be planned for your upcoming surgery.”

What is your age?

What race do you identify with?

What ethnicity do you identify with (Hispanic or Latino, Non-Hispanic or Latino, other, prefer not to say)?

What religion do you identify with?

What is your highest level of education?

What is the reason you are receiving colorectal surgery?

What is the number of previous deliveries/births you have undergone?

Have you undergone previous removal of the fallopian tubes? Yes. No. Unsure.

*(If yes, then this concludes the interview, thank patient for their time)*

Are you currently past menopause (1 year with no menstrual bleeding): Yes/No

Have you undergone previous removal of the uterus (Hysterectomy) Yes. No. Unsure.

Have you undergone previous permanent sterilization? (examples, Essure implant, tubal ligation, tubal banding or clips) Yes. No. Unsure.

Are you finished with childbearing/future fertility: Yes. No. Unsure.

Are you interested in permanent sterilization (if not menopausal)? Yes. No. Unsure.

Do you have a first or second degree family member who has had ovarian cancer (parent, sibling, child, aunt/uncle, cousin, niece/nephew)? Yes. No.

Were you aware that removal of the fallopian tubes reduces the risk of developing ovarian cancer? Yes. No.

If this was able to be offered currently, how likely are you to elect for removal of the fallopian tubes at the same time as your upcoming surgical procedure?

1- Very unlikely

2- Unlikely

3- Neutral

4- Likely

5- Very likely

If you would consider fallopian tube removal at the time of surgery, do you think there is any chance you would experience regret? Yes. No. Maybe. Would not consider (x)

If you are someone who is not interested in fertility and is not interested in removal of fallopian tubes at the time of your surgery, could you explain briefly why?

Do you have any concerns with removal of the fallopian tubes?
